# An Ambulatory Electroencephalography System for Freely Moving Horses: An Innovating Approach

**DOI:** 10.3389/fvets.2017.00057

**Published:** 2017-05-02

**Authors:** Hugo Cousillas, Martial Oger, Céline Rochais, Claire Pettoello, Mathilde Ménoret, Séverine Henry, Martine Hausberger

**Affiliations:** ^1^Université de Rennes 1, CNRS UMR 6552 – Ethologie Animale et Humaine EthoS, Rennes Cedex, France; ^2^IETR, Université de Rennes 1, UMR CNRS 6164, Rennes Cedex, France; ^3^Université de Rennes 1, CNRS UMR 6552 – Ethologie Animale et Humaine EthoS, Station Biologique, Paimpont, France

**Keywords:** electroencephalography, horses, freely moving, EEG headset, Telemetry

## Abstract

Electroencephalography (EEG) that has been extensively studied in humans presents also a large interest for studies on animal brain processes. However, since the quality of the recordings is altered by muscular activity, most EEG recordings on animals are obtained using invasive methods with deeply implanted electrodes. This requires anesthesia and can thus only be used in laboratory or clinical settings. As EEG is a very useful tool both for detecting brain alterations due to diseases or accidents and to evaluate the arousal and attentional state of the animal, it seemed crucial to develop a tool that would make such recordings possible in the horse’s home environment, with a freely moving horse. Such a tool should neither be invasive nor cause discomforts to the horse as the usual other practice which consists, after shaving the zone, in gluing the electrodes to the skin. To fulfill these requirements, we developed a novel EEG headset adapted to the horse’s head that allows an easy and fast positioning of the electrodes and that can be used in the home environment on a freely moving horse. In this study, we show that this EEG headset allows to obtain reliable recordings, and we propose an original evaluation of an animal’s “EEG profile” that allows comparisons between individuals and situations. This EEG headset opens new possibilities of investigation on horse cognition, and it can also become a useful tool for veterinarians to evaluate cerebral disorders or check the anesthesia level during a surgery.

## Introduction

Electroencephalography (EEG) has been extensively studied in humans over the last decades (1). It has been especially used in human medicine as a diagnostic tool to assess cerebral dysfunctions like epilepsy (2, 3). EEG is especially useful to characterize different levels of vigilance from the different stages of sleep to wakefulness (4). In humans, EEG recordings are mostly based on nontraumatic external electrodes placed on the head’s skin. The quality of these human EEG recordings depends on the subject’s quietness, and hence, the level of muscular activity may interfere: the subject must be quiet and avoid any movement. Adult humans can be instructed to do so.

Of course, more difficulties are, thus, encountered with animals. EEG presents also a large interest for studies of animal brain processes from basic research on sleep; attention; and awareness to applied issues, such as the impact of anesthesia, brain damages, induced or spontaneous brain diseases, and epilepsy (5–7). Although EEG recording techniques have been developed for animals, there are important constraints that may prevent to exploit the full range of possibilities offered by EEG.

Thus, in awake animals, it is almost impossible to avoid movements, and therefore, most EEG recordings in animals have been done invasively using deeply implanted electrodes (8, 9), in which case, the electrodes record directly the cortical activity (electrocorticogram), and hence, produce high-quality data. Otherwise, subdermal needle could be used, which may also induce discomfort or even pain, leaving potentially negative memories (10, 11). Such techniques have been used in equids and have allowed to characterize the EEG waves of the different stages of vigilance (4). Of course, this requires a surgery under anesthesia to implant the electrodes that can be quite traumatic. This, of course, is not adapted for “routine” use, especially in large domestic species.

Other authors have developed a noninvasive approach on awake animals: the animals are kept immobile (generally in metal stocks), the electrodes are glued on the animal’s head and any movement has negative consequences (12).

Often the horse’s forehead has to be shaved, which in the case of horses may be a problem for both esthetical reasons (owners may refuse if the EEG is for non-pathological reasons) and be a source of fear for untrained (to the procedure) horses, which limit the population that can be tested. Moreover, the affixed electrodes may, at the time of removal, create a “bad memory” of the procedure that makes the horse reluctant to accept them again on further occasions. This anticipation may also impact the quality of the EEG recordings (see further).

However, EEG is a precious tool not only for evaluating brain alterations or dysfunction due to diseases or trauma (7), but also to study the vigilance state of the animal, for testing the impact of drugs, welfare reasons (13, 14), or studies on cognition. It is then crucial to be able to use it in a field situation that is in the home environment of the horse and preferably on freely moving animals. Thus, it is absolutely necessary that the recording apparatus (1) can be used in the home environment, (2) is easy and rapid to adapt to each horse, (3) can be used on a freely moving animal (that can orient toward a stimulus), and (4) does not put more constraint on the horses than the usual gear such as halter of snaffle (i.e., rapid habituation).

In this study, we present a novel EEG headset adapted to the horse’s head that allows an easy and fast (less than 5 min) positioning of the electrodes that solves all the above-mentioned issues (15). The aim of this article is to describe this novel system and display the EEG recordings obtained. We also describe here an original approach for analyzing the EEG data through an evaluation of the relative proportion of the different types of waves. This approach allows to examine intra- and interindividual variations in the “EEG profile” within a given situation and intraindividual variations between situations. Here, we show EEG recordings obtained from five relaxed standing adult sport horses that were freely moving in their home stall over two sessions performed on two successive days.

## Materials and Methods

### Material and Technological Developments

**Figure 1:**
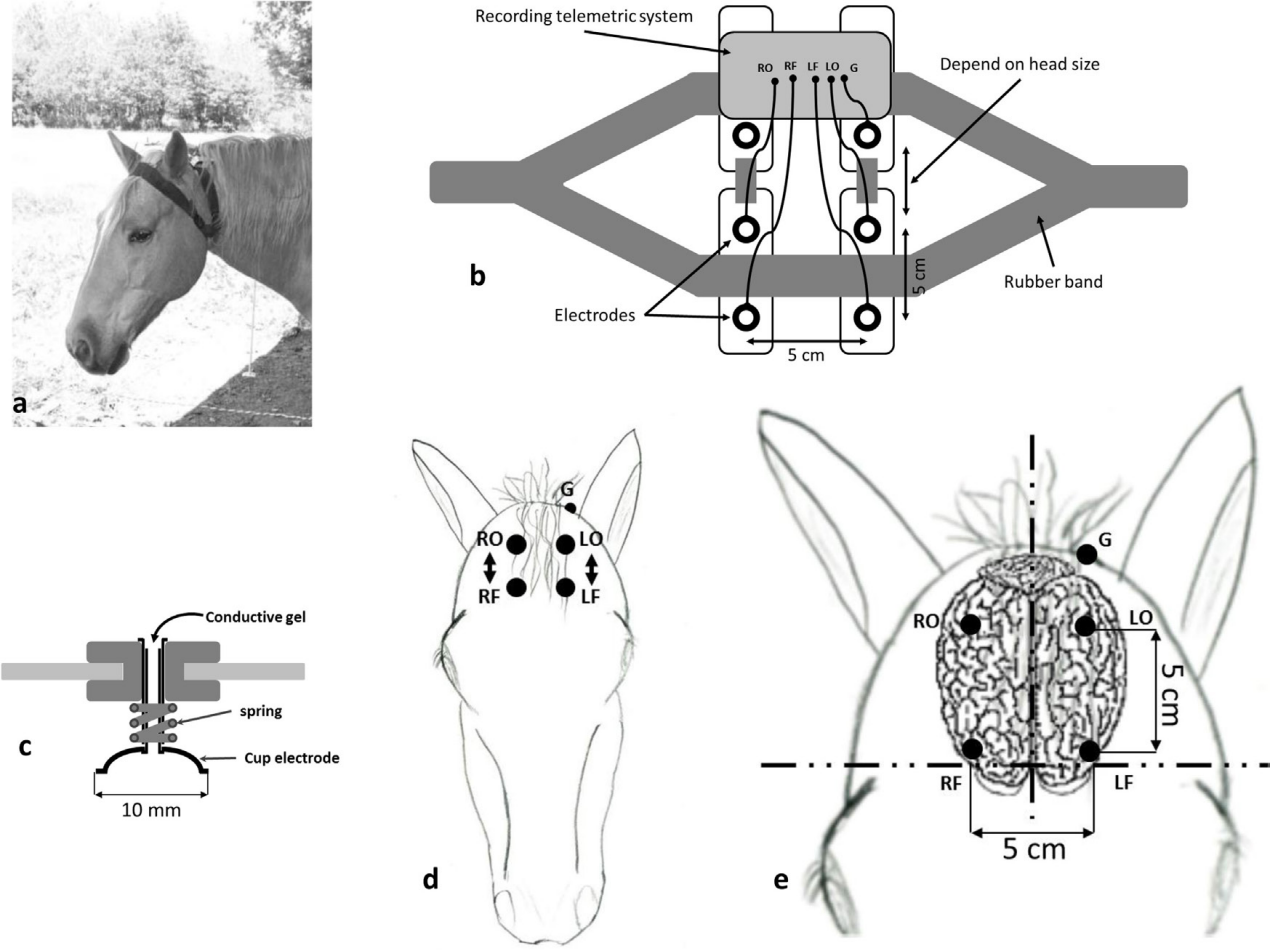
**(A)** Electroencephalography (EEG) headset, a necklace made of large rubber bands, placed on the horse’s head. **(B)** Schematic representation of the EEG headset. **(C)** Cup gold electrodes with a 10-mm diameter (DSGSAS102600 Spes Medica) mounted with a spring on a tube that can be filled with a conductive gel (NEURGEL250F Spes Medica) in order to have a good electrical contact. **(D)** Position of the electrodes, RO, right occipital; RF, right frontal; LO, left occipital; LF, left frontal; G, body ground. **(E)** The electrodes locations with the anatomical landmarks and the measurements are shown on a schematic representation of the horse brain that is located just underneath the frontal bone.

The main innovation was the EEG headset (Figure 1A; patent # R23701WO) that allowed us to place easily five electrodes on the horse’s forehead over the parietal and frontal bones. The design of this headset, a necklace made of large rubber bands, allowed a good and stable electrode contact with the horse skin and was adjustable for different head sizes (Figure 1B).

#### Electrode Positioning

The electrodes (Figure 1C) were cup gold electrodes with a 10-mm diameter similar to those used on humans (DSGSAS102600 Spes Medica). They were filled with a conductive gel (NEURGEL250F Spes Medica) in order to have a good electrical contact. They were positioned on each side of the horse forehead to allow separate hemispheric recordings (Figure 1D). On each side, the electrodes were placed on occipital and frontal positions. This electrode positioning, similar to the one used by Mysinger et al. (16), allowed us to record the differential activity between the most occipital part of the brain and the most frontal one. On an adult horse, the most rostral electrodes were positioned on a reference line located between the zygomatic processes of the frontal bones (Figure 1E). The occipital electrodes were located 5 cm backward from the frontal ones. Left and right electrodes, positioned on either side of the sagittal plane, were separated by 5 cm. The ground electrode was placed on the back of the left ear that allowed us to avoid most of the muscular artifacts. Using these anatomical landmarks allowed a precise repositioning of the electrodes on repetition with less than 2 mm differences.

#### Telemetric Recording Setup

**Figure 2:**
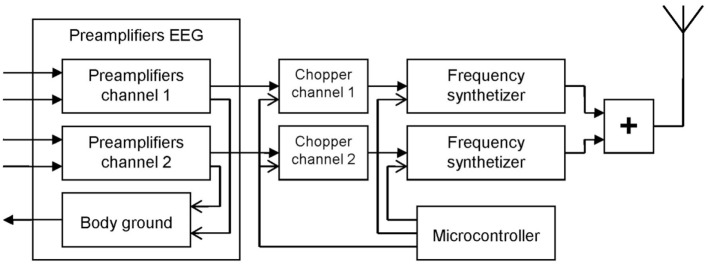
**Schematic diagram of the homemade recording telemetric system used in this study**. The electroencephalography (EEG) amplifier (left) was based on the two-way recordings “ModularEEG” from the openEEG project (http://openeeg.sourceforge.net/doc/index.html).

The electrophysiological recordings were performed, thanks to a homemade telemetric recording setup (L, 110 mm; l, 90 mm; h, 30 mm; weighting, 110 g) developed by Martial Oger (Figure 2). The homemade EEG amplifier was based on the two-way recordings “ModularEEG” from the openEEG project (http://openeeg.sourceforge.net/doc/index.html). This amplifier was connected to the telemetric radio transmitter. The whole telemetric recording setup was fixed on the headset.

Any existing telemetric system could fit with the headset. In the example described here, we used a homemade system. After being amplified the EEG signal was chopped in order to modulate two oscillators working with two nearby frequencies (around 433 MHz). The output signals of the oscillators were added and delivered to a quarter of wave whip antenna. The oscillators were both stabilized and modulated in frequency. The central frequencies of the oscillators were stabilized using a frequency synthesizer in the 50 Hz bandwidth. In order to modulate the frequency of the oscillators, the EEG signal frequencies that are too low to modulate these oscillators were shifted toward higher frequencies. Thus, the EEG signals were chopped with a period of 1 msec (1 kHz). A microcontroller was used to drive the two frequency synthesizers and to command the choppers.

To avoid wiring around the head and along the neck of the horse and to allow the animal to move freely, a homemade EEG amplifier associated with a radioemitter was also developed. With this telemetric EEG amplifier that was fixed directly on the recording headset on the head, the animal was completely detached from any monitor.

To obtain the EEG signals, we used a classical FM receiver associated with an envelope demodulator. The demodulation of the signal received gives an audio signal chopped at 1 kHz, and to obtain the EEG signals, we made an envelope demodulation of this audio signal.

The EEG signals were then recorded in a computer using a digital oscilloscope “Picoscope 2200 Series” (pico^R^ Technology) and the Picoscope Oscilloscope Software. The sampling rate was 1 kHz.

### The Animals and the Experimental Procedure Used in the Test Study

The recordings were performed at the French national equitation school of Saumur on five horses (three French saddlebred, two Anglo-Arab) aged 7–9 years (four females and one gelding), trained for competition [show jumping (*N* = 3) and eventing (*N* = 2)].

In the test study, the horses were quietly standing. Each horse was tested during two sessions of 15 min, one on the first day (D1) and the other on the following day (D2). All experiments were video-recorded using the media-recorder software that allowed to record and synchronize several videos. Behaviors were then encoded and synchronized with EEG recordings using “The Observer” software from Noldus.

#### Data Analysis

We processed all data using FieldTrip (17), a free toolbox of Matlab 7.0 (MathWorks, Natick, MA). For each horse, the videos were screened, and the periods when the animal was standing relaxed were identified. EEG signals during these time periods were visually inspected, and large artifacts, due to movements (body, head, ears), were removed. The data were then segmented into 500 ms epochs, and a second visual artifact rejection procedure was performed to exclude potential remaining artifacts. There were an average of 86.45 ± 9.05 epochs/horse/session. Then, a complex fast-Fourier transform was applied on these 500 ms epochs in which, for each frequency, outlying values ±3SD were identified, and the corresponding epoch was removed from the analysis. The FFT values for each horse were averaged separately for each session. All usual waves were present (delta, theta, alpha, beta, and gamma), but their relative weight varies according to the animal state. In the present case, we concentrated on the pertinent waves and extracted the power values of the alpha (8–12 Hz), beta (12–30 Hz), and gamma (>30 Hz) frequency bands, characteristic of awake animals. We calculated the proportion of the mean power values of these three wave types (alpha, beta, and gamma) to express these values as a percentage. Then, to evaluate the stability of the “EEG profiles” of the different individuals and over the time, the coefficients of variation (Cv = SD/mean) of these proportions were calculated. Coefficients of variations have long been shown to be a reliable way of broaching the question of the levels of variation (18).

## Results

**Figure 3:**
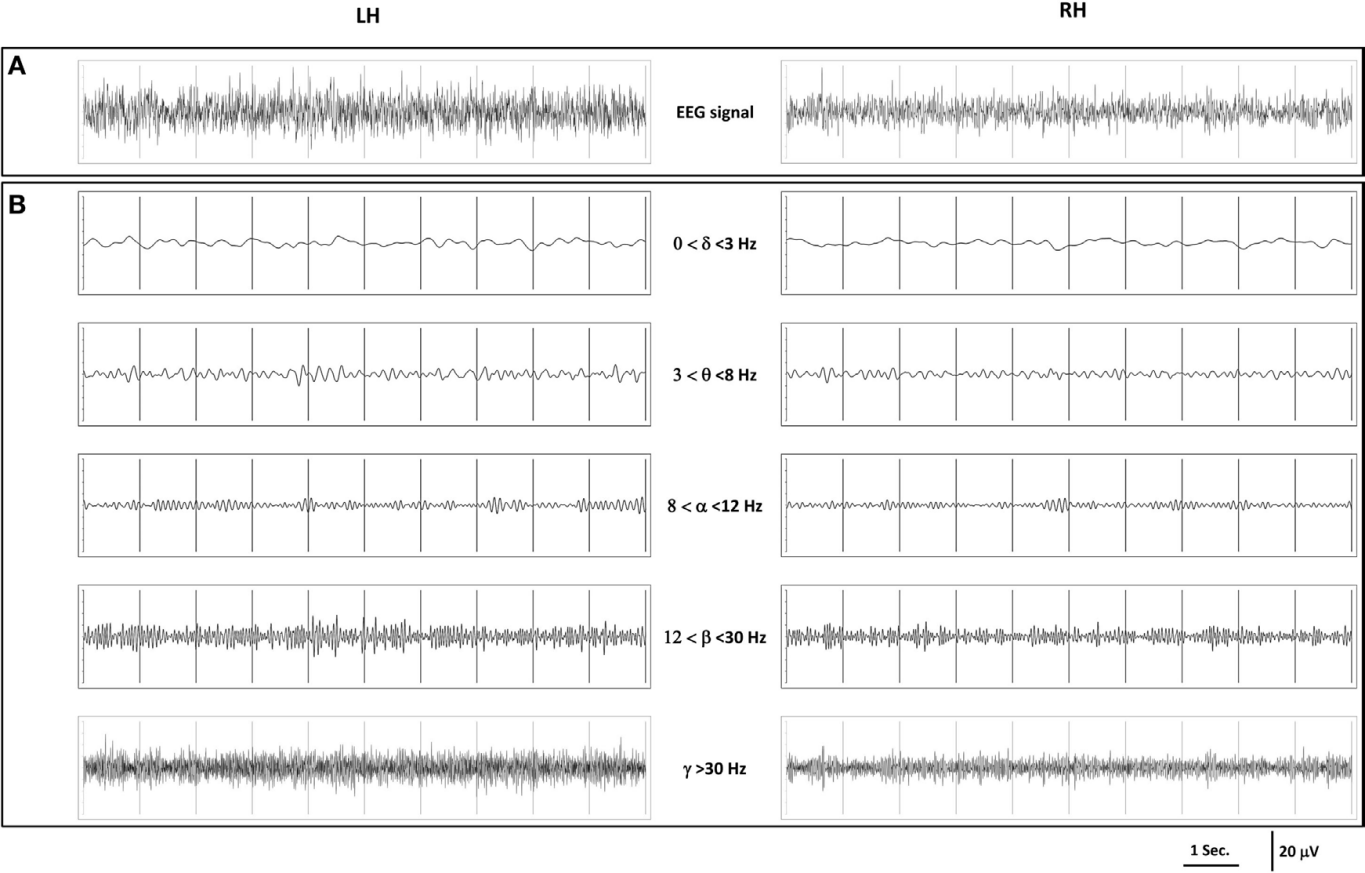
**(A)** Example of 10 s electroencephalography (EEG) recordings obtained in a relaxed standing horse; **(B)** representation of each wave range of these recordings.

The EEG recordings obtained were similar to those produced by other methods (Figure 3). This EEG, similar for all horses, was characteristic of a quiet awake state and in accordance with those obtained in previous studies (16) using external or implanted electrodes, with a clear desynchronization, low to medium voltages, and slow and more rapid waves superimposed (Figure 3A). There was no difference between hemispheres, and thus, the data were pooled for further analysis (see last part).

To disentangle the relative part of the different waves, the EEG recordings were further filtered and revealed that low frequencies (delta and theta) that are characteristic of slow wave sleep were less represented and of low amplitude (<10 μV), while three frequency ranges (among the five) dominated in all horses: the alpha waves (8–12 Hz) that are characteristic of a relaxed state, the beta (12–30 Hz), and the gamma waves (>30 Hz) that are characteristic of higher awareness state with amplitudes of 10–30 µV (Figure 3B).

**Figure 4:**
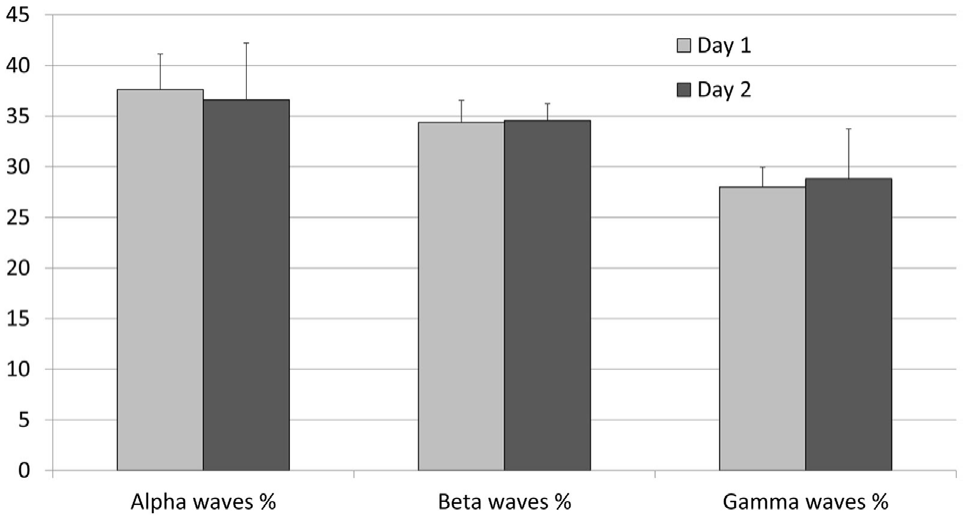
**Proportions of the power spectrum of alpha, beta, and gamma waves recorded on the five relaxed standing horses on two different recording days**. Error bars indicate mean ± SD.

We then tried to evaluate the potential interindividual variations in the EEG pattern by calculating the relative proportion of the three dominating wave ranges (Figure 4). On day 1, the proportion of alpha, beta, and gamma waves was, respectively, of 37.63% (±3.5), 34.38% (±2.19), and 27.99% (±1.97) and on day 2, 36.62% (±5.6), 34.57 (±1.66), and 28.81 (±4.92).

The interindividual variation was very low with coefficients of variations of 12.29%, 5.59%, and 12.06%. Obviously, in a same context, the EEG pattern of different horses was very similar.

Interestingly, the intraindividual variation (same individual’s day 1/day 2) was still lower with mean coefficients of variation of 6.39%, 4.69%, and 9.22% for alpha, beta, and gamma waves, respectively. It is worth noting that the proportion of beta waves appeared the most stable feature both at the inter- and intraindividual levels.

## Discussion

The novel EEG headset for EEG recordings proved to be quite adapted for recordings of freely moving animals in their home environment (15). The use of a telemetric device enabled us to avoid the usual constraints related to cabling. Typical EEG recordings were obtained for the five relaxed standing horses: the major part in the EEG recordings was composed of frequencies higher than 8 Hz. Since the recordings have been performed on awake horses, low frequencies (delta and theta waves) that are characteristic of slow wave sleep were weakly represented here. We recorded small amplitude alpha waves, which is in accordance with Williams et al.’s (12) study. All horses showed alpha waves, which is interesting as according to Mysinger et al. (16) only half of the horses presented alpha waves in their study. The authors noted that the horses that were lacking these waves were apprehensive during the recordings, which is logical because cortical arousal associated with anxiety would increase beta and decrease alpha frequencies. One reason why we observed them in all horses may be because of the conditions of recordings that differ from the abovementioned study: here, the horses were totally freely moving in their usual stall and the device was very light, whereas in the other study, they were restrained and had a heavier device to wear. For example, in equids, several authors have developed nontraumatic EEG recordings (16, 19), with the aim of studying EEG waves during anesthesia, sleep, or even epilepsy (7, 12, 20–22). The electrodes were positioned and glued on the head, a conductive paste was added. In general, the horse’s forehead was shaved to have a good contact. Long wires were used to connect the electrodes to the amplifiers, and the recording system was placed on the back of the horse. These EEG recording systems are better and easier to use than those using implanted electrodes, but they still need a relatively long preparation. Moreover, the horses were placed between bars preventing them to move their body. However, the temporal region of the horse’s head is heavily muscled and movements of the ears, the eyes, and the jaws impacted strongly the recordings, and a good part had to be left aside for these reasons. Being able to record EEG in freely moving animals in their familiar settings may, therefore, considerably change the knowledge we have of brain processing in horses.

The lower variability of intraindividual recordings shows that this headset can be placed precisely on the horse head and that it allows a precise repositioning of the electrodes.

Among the three types of waves, the beta waves were more stable. The horses were awake, quiet, and looking around, which means that attentional processes may vary as the animal is taking care or not of what is going on around it. The beta waves are characteristic of awake states, whereas the gamma waves are involved on higher awareness states and alpha waves on quiet awake states. The higher variability found on alpha and gamma waves may reflect changes on attentional processes due to environment changes.

The aim of the system we developed was as follows: (1) the system can be used in the home environment, (2) easy and rapid to adapt to each horse, (3) can be used on a freely moving animal, and (4) does not put more constraint on the horses than the usual gear. We answer all these questions: the system can be used on familiar environment, on a freely moving horse, without more constraint than the usual gear, easy and rapid to adapt on each horse and that allows reliable recordings.

This apparatus will be a precious tool for future studies aiming at evaluating, in the “field” situation, individual differences in cognitive processes (Rochais et al., in preparation) or vigilance states in different contexts such as sleep or anesthesia. For the latter, further studies will be needed to examine the reliability of data over prolonged periods of time, as this study, as a first step, was based on short-time periods.

## Ethics Statement

All procedures were in accordance with the European Communities Council Directive of 22 September 2010 (2010/63/UE) and the French law relative to the protection of the animal used in scientific experiment (Décret no. 2013-118 13 février 2013; Article R. 21488). According to these European and French laws, our experiment did not require an authorization request. Indeed, our manipulations did not cause any physical or mental pain, they consisted only of a common practice, which is the positioning of a necklace on a horse head that was freely moving in its home environment.

## Author Contributions

HC, CP, and MH conceived the EEG helmet; MO conceived the telemetric system; HC, CR, SH, and MH conceived the study and designed the experiments. HC and CR conducted experiments. MM, HC, CR, and MH analyzed the data; HC and MH wrote the manuscript, and all the authors participated in the revision of the manuscript.

## Conflict of Interest Statement

The authors declare that the research was conducted in the absence of any commercial or financial relationships that could be construed as a potential conflict of interest.
